# Causes and Biophysical Consequences of Cellulose Production by Pseudomonas fluorescens SBW25 at the Air-Liquid Interface

**DOI:** 10.1128/JB.00110-19

**Published:** 2019-08-22

**Authors:** Maxime Ardré, Djinthana Dufour, Paul B. Rainey

**Affiliations:** a Laboratoire de Génétique de l’Evolution, Ecole Supérieure de Physique et de Chimie Industrielles de la Ville de Paris (ESPCI), CNRS UMR 8231, PSL Research University, Paris, France; b Department of Microbial Population Biology, Max Planck Institute for Evolutionary Biology, Plön, Germany; Geisel School of Medicine at Dartmouth

**Keywords:** continuum field models, microbial mats, pellicle, pyoverdin, spatial structure

## Abstract

Cellulose-overproducing wrinkly spreader mutants of Pseudomonas fluorescens SBW25 have been the focus of much investigation, but conditions promoting the production of cellulose in ancestral strain SBW25 and its effects and consequences have escaped in-depth investigation through lack of an *in vitro* phenotype. Here, using a custom-built device, we reveal that in static broth microcosms, ancestral SBW25 encounters environmental signals at the air-liquid interface that activate, via three diguanylate cyclase-encoding pathways (Wsp, Aws, and Mws), production of cellulose. Secretion of the polymer at the meniscus leads to modification of the environment and growth of numerous microcolonies that extend from the surface. Accumulation of cellulose and associated microbial growth leads to Rayleigh-Taylor instability resulting in bioconvection and rapid transport of water-soluble products over tens of millimeters. Drawing upon data, we built a mathematical model that recapitulates experimental results and captures the interactions between biological, chemical and physical processes.

**IMPORTANCE** This work reveals a hitherto unrecognized behavior that manifests at the air-liquid interface that depends on production of cellulose and hints at undiscovered dimensions to bacterial life at surfaces. Additionally, the study links activation of known diguanylate cyclase-encoding pathways to cellulose expression and to signals encountered at the meniscus. Further significance stems from recognition of the consequences of fluid instabilities arising from surface production of cellulose for transport of water-soluble products over large distances.

## Abstract (precis)

This work reveals a hitherto unrecognized behavior that manifests at the air-liquid interface that depends on production of cellulose and hints at undiscovered dimensions to bacterial life at surfaces. Additionally, the study links activation of known diguanylate cyclase-encoding pathways to cellulose expression and to signals encountered at the meniscus. Further significance stems from recognition of the consequences of fluid instabilities arising from surface production of cellulose for transport of water-soluble products over large distances.

## INTRODUCTION

Surfaces are frequently colonized by microbes. Surface-associated microbes grow as dense populations/communities termed “biofilms” (1–3). Growth at surfaces provides microbes with nutrients and opportunities for cross-feeding (4, 5). For pathogens, surface colonization is often a prelude to invasion (6, 7). Microbes in high-density populations can find protection against external factors such as antibiotics and toxic agents (8). At the same time, microbes in biofilms experience intense competition for resources and can be negatively impacted by costs associated with exposure to metabolic waste products (9). For long-term survival, escape from surfaces and dispersal is crucial (10).

Primary attention has been given to colonization of solid-liquid surfaces (11, 12). This owes as much to the importance of these surfaces as it does the ease with which they can be studied. For example, colonization of abiotic surfaces can be measured by a simple histochemical assay or by microscopic observation using flow cells (13, 14). Decades of study have revealed insight into the role of adhesive factors, including polymers and proteinaceous adhesions involved in surface attachment and the regulatory pathways controlling their expression (15). A particular focus has been pathways for synthesis and degradation of the secondary signaling molecule cyclic di-GMP (16). For the most part, the precise signals activating these regulatory pathways are unclear. Moreover, the frequent use of mutants—sometimes intentionally, but often inadvertently—that constitutively overproduce adhesive factors has stymied progress in understanding many subtleties surrounding surface colonization.

Surfaces are also a feature of the interface between gas and liquid, but colonization of such surfaces has received much less attention (17–20). Air-liquid interfaces (ALIs) are of special relevance for aerobic organisms because colonization of the meniscus provides access to oxygen. While many motile aerobic bacteria display taxis toward oxygen, this alone is often insufficient to allow cells to overcome the effects of surface tension necessary to colonize the ALI. Where colonization is achieved, in the absence of mechanisms promoting buoyancy, cells must contend with the effects of gravity that become increasingly challenging with buildup of biomass.

The interface between air and liquid has further significance in that it often marks the divide between aerobic and anaerobic conditions. This has implications for surface chemistry with ensuing physiological effects for bacteria. For example, iron, an essential element, exists in the insoluble and biologically unavailable ferric form in the presence of oxygen, but is water soluble and freely available in the absence of oxygen (21). Bacteria growing within an initially resource-rich and oxygen-replete broth phase consume oxygen, and thus further growth requires access to the ALI (22). Bacteria that achieve colonization of this surface must then contend with iron-depleted conditions requiring the synthesis of siderophores (23).

To date, studies of colonization of the ALI have largely centered on genotypes that constitutively produce polymers such as cellulose (24). Often these have arisen as a consequence of selection experiments in static broth microcosms where mutants with constitutively active diguanylate cyclases (DGCs [and ensuing constitutive production of the respective polymers]) have a selective advantage that arises from capacity to form dense microbial mats (pellicles) at the ALI (20, 25–27). While such mutants have made clear the central importance of cellulose and related polymers (28), the generality of conclusions arising from the use of constitutively active mutants needs to be treated with caution (26). Desirable would be analysis of the biophysics of ALI colonization in wild-type bacteria, where regulation of polymer production is unaffected by mutation.

Almost 2 decades ago, it was reported that in well-mixed culture the fitness of a cellulose-defective mutant of Pseudomonas fluorescens SBW25 was equivalent to that of the wild type (ancestral) bacterium (24). Also reported in that study was a significant reduction in fitness of a cellulose-defective mutant in static broth culture, but the reasons were not determined. Recent observations of the growth of a cellulose-defective mutant of wild-type (ancestral) SBW25 made during the course of analyses of evolutionary convergence in polymer production by SBW25 (28) led to the realization of a subtle phenotype associated with absence of growth in the cellulose-defective mutant at the air-liquid interface. Unlike ancestral SBW25, the mutant grows exclusively within the broth phase, with ensuing negative effects of oxygen limitation responsible for its previously noted low fitness (24).

Here we seek to understand the biological role of cellulose and do so via a device that combines spectrophotometry with multiperspective time-lapse imaging. Aided by the device, we monitor surface growth, reveal the contribution made by cellulose, and show that it involves regulatory contributions from three known diguanylate cyclase-encoding regulatory pathways. The production of cellulose allows formation of a lawn of microcolonies at the meniscus that eventually coalesce into a thin film of bacteria. The mass of bacteria and cellulose generates a gravitational force that leads to Rayleigh-Taylor instability and causes bioconvection (29). One consequence of bioconvection is the rapid transport of the water-soluble iron-binding siderophore pyoverdin. A mathematical model based on partial differential equations with fluid dynamics described by the Navier-Stokes (NS) equation with Boussinesq approximation accounts for the diffusion-reaction and convection processes occurring in the microcosm.

## RESULTS

Describing microbial colonization of the air-liquid interface (ALI), although in principle straightforward, is fraught with difficulty. While advanced microscopic techniques offer possibilities to observe colonization at the single-cell level, much stands to be gained from more macroscopic perspectives, aided by low-power microscopy in conjunction with time-lapse photography.

### Device.

**FIG 1:**
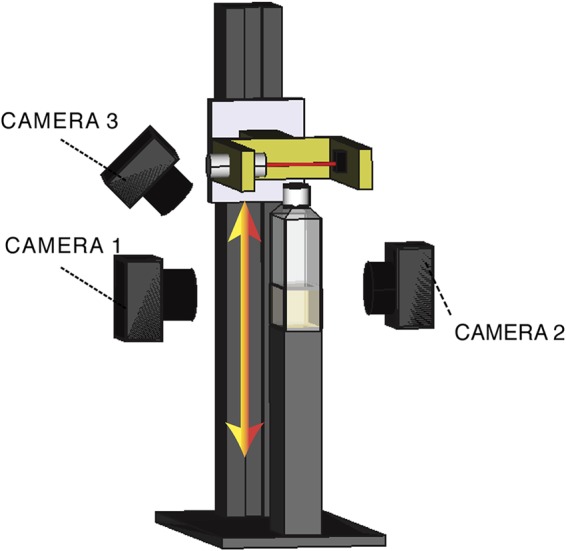
Experimental device. A polycarbonate cell culture bottle filled with 20 ml of KB and inoculated with bacteria is placed on a fixed vertical stand. The device and associated cameras are maintained within a 28°C incubator. The flask is scanned vertically every 5 min with a 600-nm laser beam with a 1-mm section. Light passing through the flask is collected by a photodiode. To obtain a measurement of the optical density in the flask along a vertical profile, the laser and linked photodiode are coupled to a motorized device that ensures smooth vertical translation. Three cameras are located around the flask. The first (camera 1) obtains a side-view image of the liquid phase of the medium using bright-field illumination. The second (camera 2), also fixed perpendicular to the flask, monitors fluorescence associated with pyoverdin (excitation of 405/emission of 450 nm). The third (camera 3) is oriented with a 45° angle and captures growth at the ALI using bright-field illumination.

To understand and measure growth of ancestral SBW25 and a cellulose-defective mutant with the *wssABCDEFGHIJ* operon deleted, SBW25 Δ*wssA–J*, a device was constructed that allows growth at the ALI and in the broth phase to be monitored from multiple perspectives (Fig. 1). It comprises three cameras: one placed perpendicular to the microcosm to record growth within the microcosm and on the undersurface of the meniscus, one mounted at a 45° angle above the ALI to capture surface growth, and one to detect the light emitted from excitation of the fluorescent signal arising from production of the iron-chelating siderophore pyoverdin. Additionally, the device incorporates a laser and corresponding photodiode to vertically scan the flask at regular (5-min) time intervals.

### Cellulose is required for colonization of the ALI.

**FIG 2:**
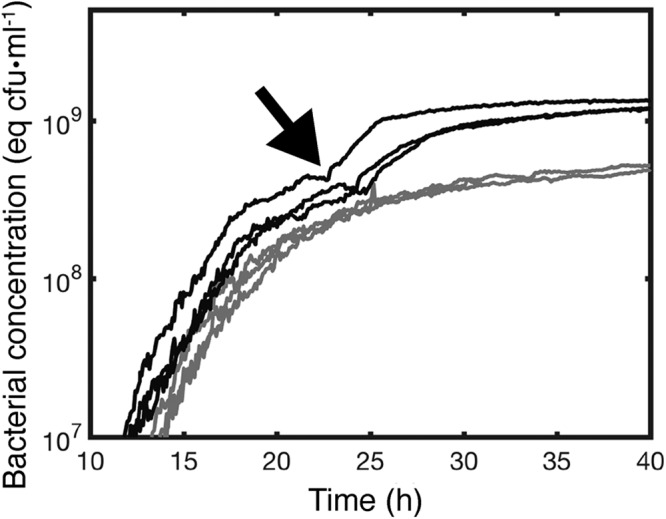
Production of cellulose maximizes growth in static broth culture. Dynamics of growth of P. fluorescens SBW25 (black lines) and P. fluorescens SBW25 Δ*wssA–J* (cellulose-negative mutant) (gray lines) in unshaken KB as determined by the scanning laser device and associated photodiode depicted in Fig. 1. Every curve is an independent experiment made in a new flask. Data are the spatial average of the optical density at 600 nm (OD_600_) obtained from scanning the vertical section of a flask. OD_600_ measurements are calibrated using direct plate counts of CFU (equivalent CFU · ml^−1^). Measurements were taken every 5 min. The arrow denotes the onset of bioconvection caused by production of cellulose that marks a secondary increase in growth. This second growth phase is absent in the cellulose-negative mutant.

Figure 2 shows the growth dynamics of ancestral SBW25 and SBW25 Δ*wssA–J* determined by the scanning laser and calibrated using direct plate counts. SBW25 Δ*wssA–J* is slower to enter exponential growth than SBW25, it grows at approximately the same rate (SBW25, 0.53 ± 0.02 h^−1^; SBW25 Δ*wssA–J*, 0.57 ± 0.03 h^−1^), but density in stationary phase is consistently lower. Notable in SBW25 at 24 h is a reproducible plateau of growth followed by a further increase and a widening of difference in cell density compared to SBW25 Δ*wssA–J* (Fig. 2). No such intermediate plateau occurs in the cellulose mutant.

**FIG 3:**
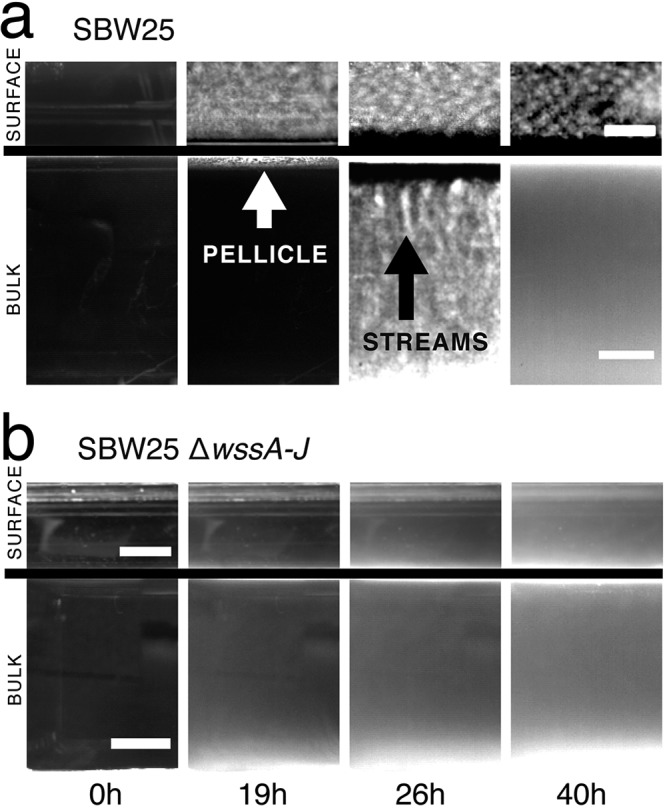
Cellulose is necessary for growth at the air-liquid interface (ALI) and results in bioconvection. Shown are bright-field images of ancestral P. fluorescens SBW25 (a) and SBW25 Δ*wssA–J* (cellulose-negative mutant) (b) taken at four time intervals. Complete movies are available as Movies S1, S2, S6, and S7 at figshare (https://figshare.com/projects/Causes_and_consequences_of_cellulose_production_by_Pseudomonas_fluorescens_SBW25_at_the_air-liquid_interface/59630). Images above the line show growth at the ALI captured using camera 3; images below the line are from camera 1 (Fig. 1). At 0 h, the medium is inoculated with ∼10^4^ cells · ml^−1^. By 19 h, the ancestral cellulose-producing genotype has formed a thin white pellicle at the ALI (visible by both cameras 1 and 3). No pellicle formation is seen in the cellulose-negative mutant, but growth is evident in the broth phase. By 26 h, in cultures of the cellulose-producing ancestral type, plumes characteristic of bioconvection stream from the ALI (pointed at by the black arrow). No evidence of mat formation or streaming is seen in SBW25 Δ*wssA–J*. By 40 h, streaming has largely ceased in the ancestral type, although growth is still apparent at the ALI. Scale bars are 5 mm. Contrast has been adjusted to highlight salient features.

Time-lapse observation of the ALI from a 45° angle in flasks inoculated with SBW25 reveals the presence of a thin film at 19 h that is more prominent at 26 h and still evident, albeit weakly, at 40 h (Fig. 3a; see Movie S1 at figshare [https://figshare.com/projects/Causes_and_consequences_of_cellulose_production_by_Pseudomonas_fluorescens_SBW25_at_the_air-liquid_interface/59630]). Beyond the 40-h time period, wrinkly spreader mutants arising within the flasks begin to grow at the ALI. In contrast, no evidence of colonization of the ALI is evident in SBW25 Δ*wssA–J* (Fig. 3b; see Movie S2 at figshare). Observations from the camera perpendicular to the flask confirmed the presence of surface growth in SBW25 (Fig. 3a) but not in the cellulose mutant (Fig. 3b). Additionally, rapid streaming was observed in the broth phase for the ancestral genotype but not for SBW25 Δ*wssA–J* (Fig. 3; see Movies S3 and S4 at figshare). The significance of this streaming dynamic is considered in detail below.

**FIG 4:**
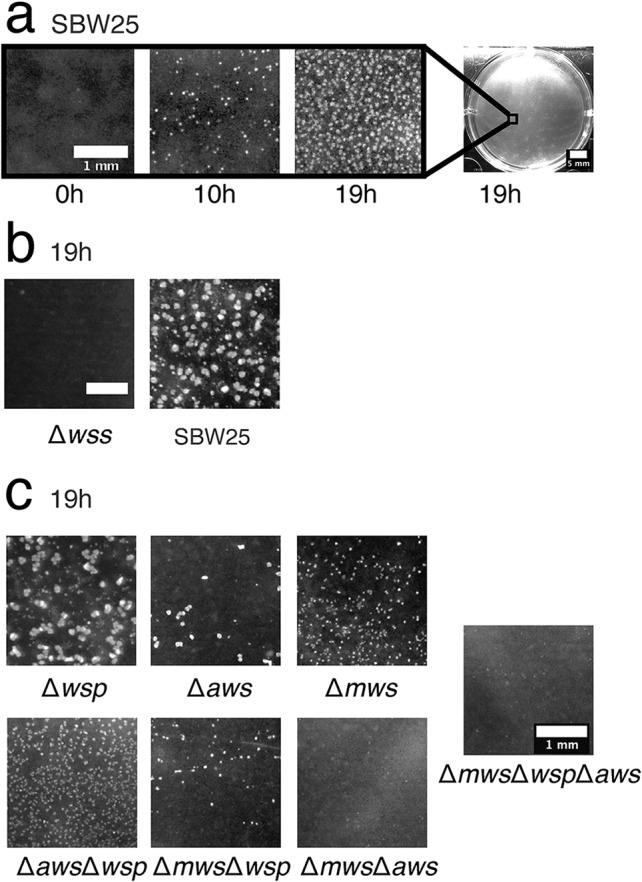
Multiple diguanylate cyclases are required for colonization of the ALI. Microcolony formation at the ALI for ancestral P. fluorescens SBW25 and a range of mutants was captured from a camera mounted directly above individual wells of a six-well tissue culture plate containing 5 ml KB. (a) The time course of microcolony formation for ancestral P. fluorescens SBW25. The complete movie S5 can be seen at figshare (https://figshare.com/projects/Causes_and_consequences_of_cellulose_production_by_Pseudomonas_fluorescens_SBW25_at_the_air-liquid_interface/59630). (b) Comparison with SBW25 Δ*wssA–J* (cellulose-negative mutant) at 19 h. (c) Patterns of microcolony formation at 19 h in mutants devoid of the Wsp (Δ*wsp*), Aws (Δ*aws*), and Mws (Δ*mws*) diguanylate cyclase-encoding pathways and combinations thereof are shown. Scale bars are 1 mm, except in the entire well in panel a, in which the scale bar is 5 mm.

Curious as to the nature of the previously unseen surface growth, we obtained high-resolution photos at hourly intervals (between 15 h and 20 h) from directly above the surface using a light source for illumination positioned at an oblique angle to the surface. No surface growth was evident for SBW25 Δ*wssA–J* (Fig. 4b), but remarkably, from the ancestral genotype, numerous microcolonies emerged from the surface of the meniscus and grew outward as if on an agar plate (Fig. 4a). By 19 h, microcolonies were seen to fall from the surface through the effects of gravity, but this was quickly followed by coalescence and collapse of the entire population of microcolonies (Fig. 4a; see Movie S5 at figshare). Interestingly, at the moment of coalescence and mat collapse, “chewing gum-like” strands suddenly appeared at the ALI, which is more characteristic of standard pellicles (20, 24, 30). This raises the possibility that cellulose is transformed from a viscous liquid to a solid by the stretching effect of gravity.

### Regulation of cellulose and ALI colonization by multiple diguanylate cyclase-encoding regulatory pathways.

Numerous studies of constitutive cellulose-overproducing mutants—the so-called wrinkly spreader (WS) types (31)—have shown the phenotype to arise primarily by mutations in the Wsp, Aws, and Mws pathways (27–32). Mutations in the negative regulators of these diguanylate cyclase (DGC)-encoding pathways result in overproduction of cyclic di-GMP, overproduction of cellulose, and formation of substantive and enduring mats at the ALI. While these findings have connected overexpression of DGC-encoding pathways to the WS phenotype, the relationship between known DGC-encoding pathways and cellulose expression in the absence of DGC-overactivating mutations has been a mystery. Recognition that ancestral SBW25 activates cellulose production at the ALI leading to microcolony formation and a frail film of cells allowed investigation of the role of Wsp, Aws, and Mws in expression of this phenotype.

A reduction in the formation of microcolonies in SBW25 Δ*wspABCDEFR*, SBW25 Δ*awsXRO*, and SBW25 Δ*mwsR* demonstrates for the first time a connection between the Wsp, Aws, and Mws pathways, the production of cellulose, and colonization/microcolony formation at the ALI (Fig. 4c) in ancestral SBW25. Surprisingly, no single pathway mutant resulted in a cellulose-defective phenotype that matched that of the cellulose-defective *wssA–J* deletion mutant (Fig. 4c). Equally surprising was that all three pathways make some contribution to colonization of the ALI (Fig. 4c). The most pronounced phenotype was associated with SBW25 Δ*mwsR*, followed by SBW25 Δ*awsXRO* and SBW25 Δ*wspABCDEFR*. A mutant lacking all three pathways was indistinguishable from SBW25 Δ*wssA–J* (Fig. 4c).

### Cellulose causes bioconvection.

**FIG 5:**
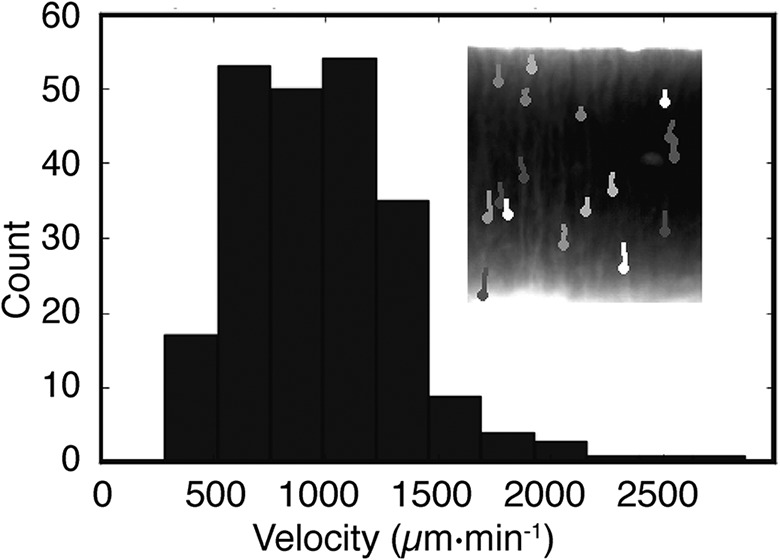
Bioconvection caused by cellulose. Time-lapse images via bright-field camera 1 (Fig. 1) capture biomass dynamics in the liquid medium. By 25 h, Rayleigh-Taylor instability generates plumes of biomass that fall from the ALI to the bottom of the flask (inset). The velocity of movement is obtained by tracking trajectories of the plumes. The frequency distribution of plume velocity reveals a mean speed ± standard deviation (SD) of 983 ± 373 μm · min^−1^.

As noted above, in microcosms inoculated with cellulose-producing ancestral SBW25, material falls in finger-like plumes that stream from the ALI (Fig. 3a). Analysis of time-lapse movies (see Movie S6 at figshare) shows plumes to be characteristic of long-range convection (Fig. 5), which arises as a consequence of instability of the interface between the cellulose-rich meniscus and the less dense broth phase beneath. The phenomenon is known as Rayleigh-Taylor instability. That cellulose is the critical component stems from the fact that the streaming plumes are evident in ancestral SBW25 but not in cultures of the cellulose-negative mutant (SBW25 Δ*wssA–J*).

Quantification of the streaming plumes shows instability at ∼25 h and continues until ∼40 h, at which point streaming ceases and the medium becomes homogeneous. The velocity of the falling plumes ranges from 500 to 2,000 μm · min^−1^ (Fig. 5). From this, it is possible to calculate the Péclet number, which defines the contribution of diffusion relative to bioconvection on the transport of water-soluble products. In this instance, the Péclet number (Pe) is ∼1,000 (calculated by multiplying the typical plume length [1 cm] by its velocity [∼1 × 10^−3^ cm · s^−1^] and then dividing by the diffusion coefficient of pyoverdin [∼1 × 10^−6^ cm^2^ · s^−1^]). The Péclet number, being greater than 1 (Pe is a dimensionless number), indicates that bioconvection is a more significant contributor to the transport of soluble products than diffusion.

### Bioconvection affects spatial distribution of extracellular products.

**FIG 6:**
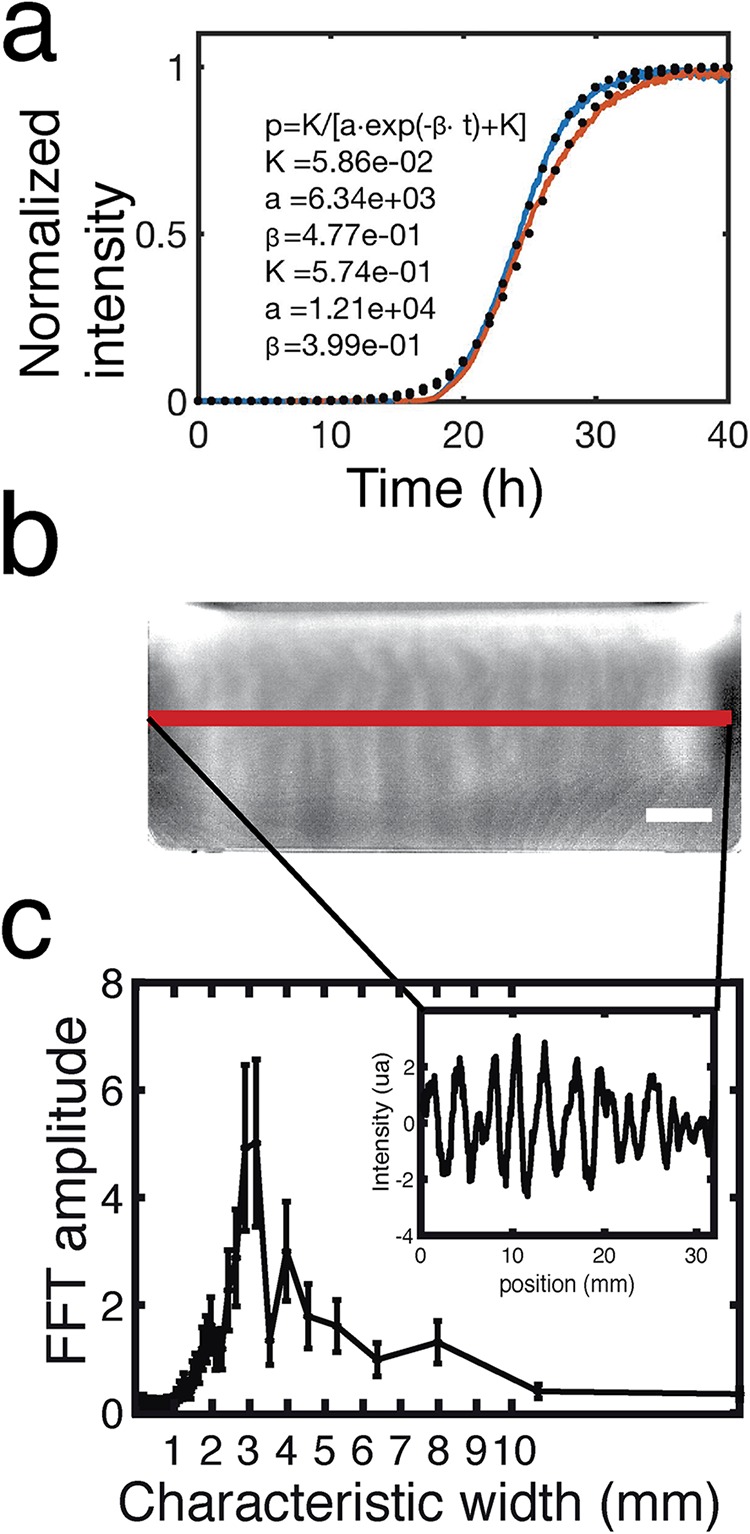
Camera 2 (Fig. 1) monitors the pyoverdin concentration in the flask by measuring fluorescence. Pyoverdin is produced primarily at the ALI. (a) The average fluorescence along the ALI increases with time like a sigmoid curve. The *ad hoc* logistic function of the inset gives the normalized intensity (*p*) as a function of the time (*t*) and the parameters of the fit (*K*, *a*, and β). The fitted curves (dotted line) adjust the experimental curves (plain line) for the estimated values of the parameters given in the inset. (b) Plumes due to Rayleigh-Taylor instability transport pyoverdin from the ALI to the liquid phase. The pyoverdin concentration is transiently higher along vertical columns that correspond to the plumes flowing from the ALI. The scale bar is 5 mm. (c) The fluorescence intensity profile along the red line (b) shows that pyoverdin is distributed with a fluctuating spatial structure (inset). Fast Fourier transformation (FFT) of the intensity profile reveals these fluctuations to have a characteristic wavelength of 3 mm.

A soluble product of relevance to P. fluorescens SBW25 in static culture is the water-soluble iron-binding siderophore pyoverdin (33). That it is fluorescent means that it is readily monitored. Figure 6a shows the average concentration of pyoverdin at the ALI as imaged via camera 2 equipped with suitable optical filters (Fig. 1). The first indication of pyoverdin production occurs at the ALI at ∼19 h and coincides precisely with the first visible stages of surface colonization where microcolonies begin to form at the meniscus (Fig. 4).

The first signs of pyoverdin production are restricted to the ALI despite the fact that at 19 h and thereafter the broth phase is turbid with growth. (Movie S3 shows turbidity in the flask, and Movie S7 shows pyoverdin in the flask [see both movies at figshare].) This is consistent with oxygen being available at the broth surface (and absent in the bulk phase due to metabolic activity), causing iron at the ALI to exist in the insoluble ferric form, leading to activation of pyoverdin synthesis solely at the ALI. The kinetics of pyoverdin production were quantified by fitting data to a simple logistic model (Fig. 6a) whose fit indicates that the underlying chemical reaction is autocatalytic and characteristic of positive feedback regulation that controls pyoverdin synthesis (33).

Visible plumes of pyoverdin (Fig. 6b) were quantified by measuring pixel intensity across a single horizontal profile (inset in Fig. 6c), as indicated by the red line in Fig. 6b. To determine the characteristic plume width (Fig. 6c), the data were analyzed by fast Fourier transformation (FFT). The transformation shows that pyoverdin is concentrated in plumes with a horizontal width of 3 mm.

### Modeling microcosm dynamics.

Surface colonization by P. fluorescens SBW25, interaction of cells with oxygen, and the ensuing effects, including bioconvection and transport of pyoverdin, draw attention to striking ecological complexity in this simplest of microcosms. To determine the match between current understanding of the interplay between biological, chemical, and physical processes and the extent to which simple biophysical mechanisms explain the observed dynamics, we constructed a model based on diffusion-reaction processes and hydrodynamics. The degree of fit between model and data stands to show how well the system is understood.

The model is based on experimental quantification of bacterial culture density, pyoverdin concentration, and fluid flow. It uses partial differential equations to account for the diffusion-reaction-convection processes within the flask. The local concentrations of bacteria, oxygen, pyoverdin, and cellulose are described as continuous fields. The liquid environment is modeled as an incompressible Newtonian fluid with a mass density that depends on the concentrations of bacteria and cellulose. Its dynamic is described by the Navier-Stokes (NS) equation using the Boussinesq approximation, in which the variations of density are neglected except in the buoyancy force (34). The coupled equations allow for inclusion of different physical interactions between the components. Details are provided in Materials and Methods.

The model was solved numerically as a means of validation. Simulations were performed on a two-dimensional grid representing a physical domain of size 1 cm^2^. The top of the domain corresponds to the ALI, with free fluid slip (liquid can move along the ALI) and no penetration boundary conditions (the meniscus cannot be deformed). The sides correspond to the lateral walls of the microcosm and the bottom of the flask. The boundary conditions on the wall allow no fluid slip (liquid cannot move along the wall) and no penetration.

**FIG 7:**
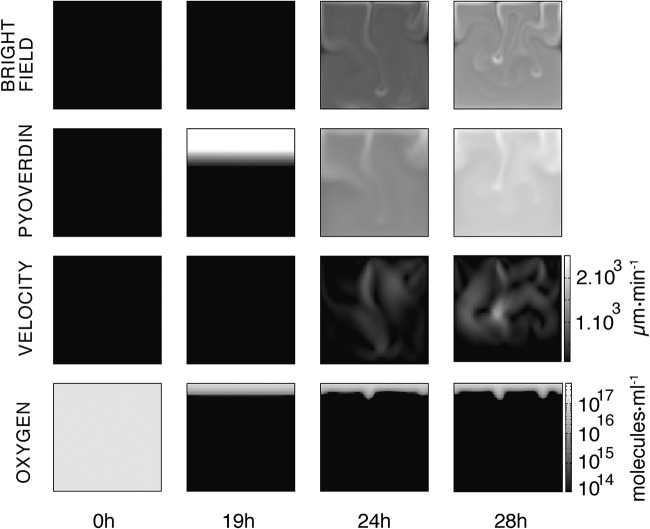
Numerical simulation of the mathematical model. Images display the dynamics of the simulated microcosm from inoculation at 0 h to 28 h. Time-resolved movies are available in Movies S8 to S11 at figshare (https://figshare.com/projects/Causes_and_consequences_of_cellulose_production_by_Pseudomonas_fluorescens_SBW25_at_the_air-liquid_interface/59630). The first row above shows the dynamics of the biomass in the bulk (bacteria and cellulose) as if observed with bright-field illumination 1 (Fig. 1). In the experiments, at 24 h plumes concentrated in biomass flow are evident in the liquid phase. The second row shows the concentration of pyoverdin in the liquid phase. The plumes transport pyoverdin into the bulk phase. The third row shows the dynamics of liquid velocity. When bioconvection is activated, fluid flow is of the order of 1,000 μm · min^−1^, which is consistent with the measurements shown (Fig. 5). The fourth row shows the dynamics of oxygen concentration. Soon after inoculation, oxygen in the bulk phase is eliminated due to metabolic (oxygen-consuming) activities of bacteria. The supply of oxygen at the ALI combined with growth of bacteria and production of cellulose means a gradient of oxygen 2 to 3mm into the liquid. Images at 24 and 28 h show oxygen transport from the ALI before consumption by bacteria in the liquid phase. The square images are 1 cm^2^, and contrast is identical across each row.

The results of the simulation are shown in Fig. 7 (see Movies S8 to S11 at figshare) and closely reproduce the dynamics observed in microcosms. Bacteria replicate and consume oxygen until growth saturates at ∼3 × 10^8^ CFU · ml^−1^. At 16 h, oxygen is available at the meniscus and in a single-millimeter layer immediately below the ALI. Also at 16 h, pyoverdin production begins; at 19 h, the first indication of cellulose production becomes visible, resulting in an increase in density of the surface layer. Soon after, cellulose-laden regions begin to form descending plumes marking the onset of Rayleigh-Taylor instability. Plumes flow from the ALI to the bottom of the flask at a speed of ∼1,000 μm · min^−1^. This is in accord with experimental observations. Additionally, plumes serve to transport pyoverdin (over a millimeter scale) and oxygen, which penetrates several millimeters into the liquid phase. Robustness of the model to changes in parameter settings was assessed by performing six simulations over a range of parameter values. Changes to *c*_0_ and o* made minimal difference over multiple orders of magnitude. Changes of 1 order of magnitude in the values of b* and ρ*_c_* eliminated bioconvection, which is expected given that these parameters are directly proportional to the mass term in the Navier-Stokes equation. Alterations to parameters V* and γ changed the dynamics of the system, leading to a delay in the onset of bioconvection. The results are shown in a supplemental data file (“supplementaryFile12” at figshare).

## DISCUSSION

The interface between liquid and air defines a niche of significance for many bacteria (4). For aerobic organisms, it is an environment replete with oxygen, it offers opportunities for unfettered surface spreading that may aid dispersal and, indirectly, may allow rapid colonization of solid surfaces; colonization of the ALI may also allow bacteria to escape grazing by solid-surface-associated predators. Despite its ecological relevance, the mechanisms and consequences of surface colonization are poorly understood.

For more than 2 decades, studies of evolution in experimental microcosms have drawn attention to adaptive mutants of P. fluorescens SBW25 that specialize in colonization of the ALI (24, 25, 31, 35). These mutants, which constitutively overproduce cellulose as a consequence of DGC-activating mutations (26–28, 30, 32, 36), reap a significant adaptive advantage in static broth microcosms because of their ability to grow at the ALI and thus their access to oxygen. Largely unknown, however, has been the ecological significance of cellulose in the ancestral type and, more generally, the role of cellulose in the natural environment. Impeding progress has been the fact that cellulose production is not evident on standard agar plate culture, and neither is it produced in shaken broth culture. In the absence of a phenotype *in vitro*, it is difficult to make progress.

Nonetheless, several previous studies have indicated environmental relevance: Gal et al. (37) showed a cellulose-defective mutant to be significantly less fit than the ancestral type in assays of plant colonization, and Giddens et al. (38) showed the cellulose-encoding *wss* operon to be specifically activated on plant root surfaces. Koza et al. showed that addition of metals, including iron and copper, to King’s medium B (KB) caused induction of a mucoid cellulose-containing agglomeration at the ALI (39). Perhaps the most significant finding, but at the time overlooked, was from competitive fitness assays between ancestral SBW25 and a *wss*-defective mutant performed in shaken and unshaken microcosms (24): in shaken culture, the fitness of the cellulose-defective mutant was no different from that of the ancestral type, but in unshaken culture, the mutant was significantly less fit. Here, prompted by recent observation of the poor growth in unshaken culture of SBW25 Δ*wssA–J* (28), combined with new tools of observation, we have come a step closer to understanding the biological significance of bacterial cellulose production.

Apparent from use of the device shown in Fig. 1 is that ancestral SBW25 activates cellulose production in static broth culture and that polymer production allows cells to break through the meniscus and, remarkably, grow transiently as microcolonies on the surface. In the absence of cellulose production, cells are unable to penetrate the ALI and fail to reap the growth advantage that comes from a plentiful supply of oxygen (Fig. 2). Just how cellulose enables bacteria to break through the ALI is unclear. One possibility is that the polymer changes viscosity and this alone is sufficient to propel bacteria through the meniscus. Another possibility is that the polymer alters surface charge and altered electrostatic properties of the cells affect interactions with the surface (40).

Also unknown is the signal or signals that lead to activation of cellulose production. What is clear is that known DGC-encoding pathways are necessary to transduce effects through to cellulose production. The fact that three pathways all contribute to different extents points to complexity in the mapping between DGCs and the cellulosic target (41). It is tempting to suggest that the signal is oxygen, but this seems unlikely because it is incompatible with the previous finding that SBW25 and a cellulose-defective mutant are equally fit in an oxygen-replete environment (24). Our suspicion is that the signal stems from some physical attribute of the ALI, possibly surface tension and Marangoni forces arising as a consequence of evaporation or production of surfactant—a subject that received momentary attention almost a century ago (42–44).

The ecological significance of the behavior is unknown. Assuming our observations are relevant to the natural environment and not just to laboratory culture, then one possibility is that cells use cellulose to colonize the ALI of water films on plant roots/leaves (the natural environment of SBW25 [45]) and use this environment to aid rapid and unimpeded dispersal. An additional benefit may then accrue on drying when the dispersed bacteria are brought back in contact with a solid substrate. One suggestive finding though that the growth extending up and out of the liquid surface may hint at a more complex and as-yet-unrecognized behavior is the involvement of three DGC-encoding pathways. Why involve three pathways to regulate cellulose production when one would seem to suffice?

As colonization of the surface begins to saturate, the heavier material on top becomes unstable and collapses in plumes typical of Rayleigh-Taylor instability. That such behavior occurs is consistent with the thesis that cellulose is produced only at the meniscus and is not evenly distributed throughout the broth phase. Numerous consequences arise from the ensuing bioconvection, one of which is the rapid transport of water-soluble products. Our particular attention has been on the fluorescent molecule pyoverdin, which by virtue of association with cells, is rapidly mixed from the point of production (the ALI) through the entire broth phase of the microcosm. Bioconvection additionally alters the chemical status of the environment, not only through mixing of extant products but also through effects wrought by enhanced transport of oxygen.

Transport of pyoverdin has particular significance in light of a previous analysis of SBW25 populations propagated in static KB culture of an extended period (23, 46). Common mutant types that rose to prominence harbored mutations that abolished pyoverdin production. The evolutionary advantage of these mutants stemmed not from scavenging of pyoverdin (akin to “cheating”), but from avoidance of the cost of producing pyoverdin when it was not required (23). That pyoverdin is not required in the broth phase (because lack of oxygen means iron exists in the soluble ferrous state) is evident from time-lapse movies (see Movie S7 at figshare [https://figshare.com/projects/Causes_and_consequences_of_cellulose_production_by_Pseudomonas_fluorescens_SBW25_at_the_air-liquid_interface/59630]) where pyoverdin production is initiated exclusively at the ALI. However, upon reaching the point of Rayleigh-Taylor instability, bioconvection due to cellulose rapidly transports pyoverdin into the broth phase, where in complex with iron, it serves to positively activate transcription of pyoverdin synthetic genes (47)—even though pyoverdin is not required by broth-colonizing cells.

Imaging of cultures as reported here draws attention to the complexity and interdependence of biological, chemical, and physical processes. A primary goal of the modeling exercise was to see just how far physical descriptions of measured phenomena such as plume velocity, bacterial density, and pyoverdin concentration could account for observed dynamics. Similar approaches have been taken previously in analysis of microbial systems (48, 49). Specifically, our model shows how dynamic processes occurring in the liquid can be affected by biofilm formation at the ALI. It also reveals how proliferation of biomass affects the production and transport of pyoverdin. Additionally it accounts for physical transport of water-soluble products and the relative contributions of diffusion versus bioconvection to this process.

The model generates results consistent with cellulose production at the ALI being sufficient to generate Rayleigh-Taylor instability and initiate fluid movement. The specific mechanisms in the model involve the imbalance between the force of mass repartition in the fluid and the damping force of viscosity. The model also supports hypotheses concerning the critical role of cellulose in bioconvection: numerical resolution of the model showed plumes to have a velocity of ∼1,000 μm · min^−1^, as observed in the experiment. Integrity of plumes—often tens of millimeters in length—is also explained by the model and arises from the fact diffusion is a minor contributor to fluid dynamics relative to the effects of bioconvection. A further insight concerns the ability of bioconvection to mix oxygen into the top few millimeters of the broth phase at a rate that is greater than its consumption. All these effects follow from the Rayleigh-Taylor instability wrought by the production of cellulose at the meniscus.

Together, the findings of this study have shed new light on the role of cellulose—a widespread microbial product (50)—in colonization of the ALI. Previous work has drawn attention to cellulose as an adhesive substance affecting the relationship between bacteria and solid surfaces (19, 51–53), but these findings stem from study systems that do not provide an opportunity for ALI colonization and perhaps by design even select mutants that overexpress cellulose and thus mislead as to ecological significance. This stated, cellulose may play different ecological roles in different organisms and under different conditions. Nonetheless, recognition that production of a polymer can modify an environment, thus significantly changing the relationship between the organism and its environment—and the environment in a more general sense—has implications for understanding a range of environments and processes affected by ALI biofilms, such as those encountered in sewage treatment plants, marine and freshwater systems, and terrestrial environments where transient films of moisture exist in soil pores and on plant surfaces. It also raises intriguing possibilities for future research on the importance of surface tension as a cue eliciting phenotypic responses in bacteria.

## MATERIALS AND METHODS

### Bacterial strain and growth conditions.

The ancestral strain of P. fluorescens SBW25 was isolated from the leaf of a sugar beet plant at the University of Oxford farm (Wytham, Oxford, United Kingdom [54]). The Δ*wssA–J* strain had the entire *wssABCDEFGHIJ* operon (PFLU0300 to PFLU0309) deleted in the ancestral background and comes from Lind et al. (28). The Δ*wsp*, Δ*aws*, and Δ*mws* variants were previously constructed by a two-step allelic-exchange strategy (27).

Strains were cultured in King’s medium B (KB) (55) at 28°C. KB contains (per liter) 20 g Bacto proteose peptone no. 3 (BD ref211693), 10 g glycerol, 1.5 g K_2_HPO_4_, and 1.5 g MgSO_4_·7H_2_O. To monitor bacterial dynamics in experimental flasks, bacteria were precultured in KB overnight, centrifuged (6,000 rpm/3,743 relative centrifugal force [rcf]) for 4 min, and resuspended in fresh KB. The optical density (OD) of suspended cultures was adjusted to an OD at 600 nm (OD_600_) of 0.8 and stored in 20-μl aliquots containing 10 μl of cultures of 0.8 OD unit and 10 μl of 60% (vol/vol) autoclaved glycerol. The aliquots were conserved at −80°C.

To establish each experiment, a rectangular flask (Easy Flask, 25 cm^2^; Nunc) was filled with 20 ml of KB medium. Twenty microliters of stock culture at −80°C was then thawed and inoculated in the KB at a final dilution of approximately 10^4^ CFU · ml^−1^. The flask was positioned in the setup shown in Fig. 1 and incubated at 28°C in an IGS60 Heratherm static incubator.

### Experimental setup to measure the dynamics of unshaken bacterial culture.

The setup was designed and built to perform custom measurements; details are available from the authors upon request. The device comprises a laser photodiode alignment to measure optical density in the flask and three cameras to observe the ALI as well as the biomass and the pyoverdin in the liquid phase.

To measure the optical density of the liquid phase, a vertical profile was obtained by scanning with a laser photodiode detector mounted on a lifter. A plastic piece that was produced by a 3D printer on a Stratasys Fortus 250 in ABS (yellow on Fig. 1) joined the laser photodiode to a carriage that was free to slide on a vertical rail (Ingus TS-01-15/TW-01-15) driven by an M10 cage bolt coupled to an M10 threaded rod. This ensured a precise vertical and horizontal positioning of the laser photodiode alignment. The thread rod was smoothly rotated using a 7.2-Vcc motor. An L293D power switch controlled by an Arduino board Mega 2560 directed rotation of the motor. The thread rod rotation angle was measured with an optical encoder, HEDS5500 500CPR. Ultimately, this allowed measurement of the vertical position of the laser beam with a resolution of ∼3 μm.

The photodiode was from Thorlabs (FDS1010), and the laser was an HLM1230 of wavelength 650 nm and power 5 mW. To ensure that the laser did not harm bacteria, the light was attenuated by a NE520B (Thorlabs) neutral-density filter of OD = 2. The optical density of the culture was evaluated by measuring the photocurrent produced by the laser hitting the photodiode after it went through the flask. To ensure linearity between the intensity of light hitting the photodiode and its conversion in photocurrent, the photodiode was polarized in inverse with 5 V provided by an LM4040 electronic component. The photocurrent was estimated by measuring the voltage of a 437% ± 5% kΩ resistor mounted in serial with the photodiode. The voltage was monitored by the Arduino Mega 2560 board encoding a 0- to 5-V analogic input on 10 bits, giving a resolution of 5 mV. After subsequent calibration, the signal acquired by the system allowed estimation of the bacterial concentration in the flask within a range of 10^7^ to 5·× 10^9^ cells·ml^−1^.

Synchronized with the laser photodiode were three cameras: a uEyeLE USB2.0 camera, a 1/2 CMOS monochrome sensor, and a 1,280- by 1,024-pixel camera equipped with a CMFA0420ND 4-mm 1/2-in. lens. The first camera (Fig. 1) records a bright-field image of the vertical side view of the culture medium. The second camera is equipped with a band-pass optical filter at 470 ± 10 nm (Thorlabs FB470-10). It takes a side view of the culture medium. During its acquisition, a 405-nm laser (405MD-5-10-1235) illuminates the flask to excite pyoverdin fluorescence. The third camera takes a bright-field image of the ALI with an angle of ∼45°. Acquisition of optical density data and photos was synchronized using a master script written in Python that recorded the data produced by the Arduino board and saved the photos taken by the cameras.

### Colonization of the ALI.

To observe the effect of Δ*wsp*, Δ*aws*, and Δ*mws* mutations on ALI colonization (Fig. 4), we used Greiner Bio-One 657160 six-well plates filled with 8 ml of KB. Each well was inoculated from glycerol stocks. The 6-well plates were incubated at 28°C without shaking. Pictures were taken with a Nikon D7000 camera equipped with an AF-S DX Nikkor 18- to 105-mm f/3.5- to 5.6-G ED VR objective.

### The advection-diffusion-convection model.

The model uses six fields to describe the system: the vector field of the fluid vorticity (ω), the scalar field of the fluid stream function (Ψ), and the scalar fields of bacterial (*b*), oxygen (*o*), cellulose (*c*), and pyoverdin (*p*) concentrations. We also use a derived vector field that represents the velocity of the fluid (*u*). The model is valid for a three-dimensional space, but we estimated its validity in a two-dimensional space in order to reduce the time of numerical computation. That is why we choose a fluid description in term of vorticity (ω) and the stream function (Ψ). This description gives two advantages for the numerical resolution of the model. First, the equation of the fluid incompressibility is solved by construction; second, the calculation of the vorticity vector can be reduced to the calculation of a simple scalar field (for more detail, see reference 34).

With the six fields given above come six partial differential equations that describe their dynamics. The first equation is related to the stream function. This scalar field is calculated by a Poisson equation

(1) ΔΨ=−ω

where Δ is the Laplace operator. The second equation deals with the vorticity reduced to a simple scalar field. Its dynamics can be derived from the Navier-Stokes (NS) equation:

(2) $$\frac{\partial \omega }{\partial t}+\left(\overset{\to }{u}\cdot \overset{\to }{\nabla }\right)\omega =\nu \Delta \omega -g\frac{\partial }{\partial x}\left(\frac{\rho }{\rho_{0}}\right)$$

The left side of NS is the Lagrangian derivative of the vorticity. The right side contains damping of the vorticity by the viscosity, ν, and a gravity term traduces the generation of vorticity due to the uneven spatial repartition of the mass density, ρ, relative to the density of the fluid medium, ρ_0_. The operator $\left(\overset{\to }{u}\cdot \overset{\to }{\nabla }\right)$ is the convective derivative. In the equation, the local mass density, ρ, takes into account the mass density of the liquid medium, ρ_0_, bacteria, ρ*_b_*, and cellulose, ρ*_c_*. Hence we consider the local mass density (ρ) as the sum of the mass contribution of the liquid medium, the bacteria and the cellulose. Explicitly the equation for ρ is

$$\rho =\rho_{0}+\Phi_{b}\left(\rho_{b}-\rho_{0}\right)+\Phi_{c}\left(\rho_{c}-\rho_{0}\right)$$

where Φ*_b_* is the local volume fraction of the bacteria and Φ*_c_* is the local volume fraction of the cellulose.

To calculate the dynamics of the concentrations of bacteria (*b*), cellulose (*c*), and pyoverdin (*p*), we write a diffusion-reaction-convection equation.

To write the third equation dealing with bacteria, we make several assumptions:

(3) $$\frac{\partial b}{\partial t}+\left(\overset{\to }{u}\cdot \overset{\to }{\nabla }\right)b=D_{b}\Delta b+\delta b\left(1-\frac{b}{b_{\text{sat}}}\right)$$

Bacteria grow exponentially until they reach the saturation, *b*_sat_, measured experimentally (Fig. 2), and bacteria consume oxygen that is dissolved into the liquid.

The left-hand side of the bacterial equation is the Lagrangian derivative applied to *b*. The right-hand side contains a diffusive term that takes into account the random motility of bacteria with a diffusion coefficient, *D_b_*, and an exponential growth term with a rate, δ, that goes to zero when the concentration reaches the *b*_sat_ value.

The fourth equation describes the dynamics of the oxygen (*o*) field. Bacteria consume the oxygen at rate γ:

(4) $$\frac{\partial o}{\partial t}+\left(\overset{\to }{u}\cdot \overset{\to }{\nabla }\right)o=D_{o}\Delta o-\gamma b\Theta (o)$$

The coefficient of diffusion is *D_o_*. Oxygen consumption goes to zero when there is no more oxygen. This is ensured by multiplying the consumption term by the Heaviside function, Θ(*o*), which is 1 when *o* is above zero, but zero otherwise.

The fifth equation assumes that the cellulose is produced with an exponential rate (α) as long as the oxygen concentration is higher than o* and the concentration of bacteria is higher than b*. Additionally, cellulose production saturates when *c* tends to 1, its maximal value. The equation is:

(5) $$\frac{\partial c}{\partial t}+\left(\overset{\to }{u}\cdot \overset{\to }{\nabla }\right)c=D_{c}\Delta c+\alpha c\left(1-c\right)\Theta (b-b*)\Theta \left(o-o*\right)$$

The sixth equation describes the dynamics of pyoverdin production. Provided that the local concentration of oxygen is sufficiently high, bacteria produce pyoverdin according to the autocatalytic synthesis measured experimentally (Fig. 5D) with rate β. The equation is

(6) $$\frac{\partial p}{\partial t}+\left(\overset{\to }{u}\cdot \overset{\to }{\nabla }\right)p=D_{p}\Delta p+\beta p\left(1-p\right)\Theta (o-o*)$$

Here, pyoverdin production goes to zero when the oxygen concentration is below o* by multiplying the production term by a Heaviside function, Θ(*o* − o*).

Finally, to calculate the fluid velocity, we use the derivative of the stream function, where *u_x_* and *u_y_* stand for the horizontal and vertical components, respectively, of the fluid velocity (*u*):

$$u_{x}=\frac{\partial \Psi }{\partial y},\;u_{y}=-\frac{\partial \Psi }{\partial x}$$

### Numerical simulations.

We used a finite difference method to solve the coupled reaction-diffusion-convection equations (56). The simulation was performed on a Linux system: Debian 4.9.51-1, gcc 6.3.0. The hardware CPU was an Intel Core i7-7700K at 4.2 Ghz with 16 GB RAM. The parameters used in the simulation displayed in Fig. 7 are listed in Table 1.

**TABLE 1:** Parameters, values, and references of data used for the model

Parameter	Symbol	Value	Unit	Source
Time step	Δ*t*	10^−3^	s	*Ad hoc*
Spatial step	Δ*x*	10^−4^	m	Constrained by CFL numerical condition (34)
Grid size, *N* × *N*	*N*	100		
Fluid dynamics viscosity	η	8.9 × 10^−4^	kg · m^−1^ · s^−1^	61
Bacterial vol	*V_b_*	3 × 10^−18^	m^3^	Exptl evidence
Water mass density	ρ_0_	0.995 × 10^3^	kg · m^−3^	61
Bacterial mass density	ρ*_b_*	1.193 × 10^3^	kg · m^−3^	57
Cellulose mass density	ρ*_c_*	1.5 × 10^3^	kg · m^−3^	*Ad hoc*
Maximal bacterial concn	*b*_sat_	3 × 10^14^	Cells · m^−3^	Exptl evidence
Initial concn of bacteria	*b*_0_	2 × 10^10^	Cells · m^−3^	Exptl settings
Diffusion coefficient of bacteria	*D_b_*	10^−10^	m^2^ · s^−1^	58
Diffusion coefficient of cellulose	*D_c_*	5 × 10^−11^	m^2^ · s^−1^	*Ad hoc*
Diffusion coefficient of oxygen	*D_o_*	10^−9^	m^2^ · s^−1^	59
Diffusion coefficient of pyoverdin	*D_p_*	3 × 10^−10^	m^2^ · s^−1^	Exptl evidence (data not shown)
Initial oxygen concn	*o*_0_	1.5 × 10^23^	Molecules · m^−3^	
Initial normalized pyoverdin concn	*p*_0_	3.36 × 10^−5^		Exptl fit
Initial normalized cellulose concn	*c*_0_	10^−8^		*Ad hoc*
Production of pyoverdin	β^−1^	1.22 × 10^−4^	s	Exptl fit
Production of cellulose	α^−1^	10^−3^	s	*Ad hoc*
Growth rate of bacteria	δ	0.53	h^−1^	Exptl fit
Oxygen consumption/bacterium/s	γ	10^6^	Molecules · cell^−1^ · s^−1^	49
Vol of cellulose produced/bacterium	V* = 10 · *V_b_*	3 × 10^−17^	m^3^	60
Minimal bacterial concn for cellulose production	b* = *b*_sat_/3	10^14^	Cells · m^−3^	Exptl evidence
Boundary condition of oxygen at top	*o*_0_	1.5 × 10^23^	Molecules · m^−3^	49
Acceleration of gravity	*g*	9.81	m · s^−2^	
Minimal oxygen concn for cellulose and pyoverdin production	o*	*o*_0_ × 10^−1^	Molecules · m^−3^	*Ad hoc*
